# Action Observation Treatment for Upper Limb Rehabilitation in Patients With Stroke: Protocol for a Multicenter Randomized Controlled Trial

**DOI:** 10.2196/42094

**Published:** 2023-04-20

**Authors:** Peppino Tropea, Francesco Infarinato, Irma Sterpi, Marco Ottaviani, Paola Antoniotti, Paola Romano, Michela Picardi, Michela Goffredo, Riccardo Re, Sanaz Pournajaf, Agnese Seregni, Antonio Caronni, Marco Franceschini, Massimo Corbo

**Affiliations:** 1 Department of Neurorehabilitation Sciences Casa di Cura Igea Milan Italy; 2 Rehabilitation Bioengineering Laboratory IRCCS San Raffaele Roma Rome Italy; 3 Neurorehabilitation Research Laboratory IRCCS San Raffaele Roma Rome Italy; 4 Department of Neurorehabilitation Sciences IRCCS Istituto Auxologico Italiano Ospedale San Luca Milan Italy; 5 San Raffaele University Rome Italy

**Keywords:** action observation treatment, stroke rehabilitation, upper limb kinematics, EEG, electroencephalography, qEEG, quantitative electroencephalography

## Abstract

**Background:**

In the last few years, new noninvasive strategies have emerged as rehabilitative treatments for patients with stroke. Action observation treatment (AOT) is a rehabilitation approach based on the properties of the mirror neuron system with a positive impact on modifying cortical activation patterns and improving the upper limb kinematics. AOT involves the dynamic process of observing purposeful actions with the intention of imitating and then practicing those actions. In recent years, several clinical studies suggested the effectiveness of AOT in patients with stroke to improve motor recovery and autonomy in activities of daily living. However, a deeper knowledge of the behavior of the sensorimotor cortex during AOT seems to be essential.

**Objective:**

The aim of this clinical trial, conducted in 2 neurorehabilitation centers and in patients’ homes, is to investigate the effectiveness of AOT in patients with stroke, confirming the translational power of a tailored treatment. Particular emphasis will be placed on the predictive value of neurophysiological biomarkers. In addition, the feasibility and impact of a home-based AOT program will be investigated.

**Methods:**

A 3-arm, assessor-blinded, randomized controlled trial will be performed by enrolling patients with stroke in the chronic stage. A total of 60 participants will be randomly allocated to receive 15 sessions of AOT with different protocols (AOT at the hospital, AOT at home, and sham AOT), 3 sessions per week. The primary outcome will be assessed using the Fugl-Meyer Assessment-Upper Extremity scores. Secondary outcomes will be clinical, biomechanical, and neurophysiological assessment.

**Results:**

The study protocol is part of a project (project code GR-2016–02361678) approved and funded by the Italian Ministry of Health. The study began with the recruitment phase in January 2022, and enrollment was expected to end in October 2022. Recruitment is now closed (December 2022). The results of this study are expected to be published in spring 2023. Upon completion of the analyses, we will examine the preliminary effectiveness of the intervention and neurophysiological outcomes.

**Conclusions:**

This study will be used to evaluate the effectiveness of 2 different AOT scenarios (ie, AOT at the hospital and AOT at home) in patients with chronic stroke and to assess the predictive value of neurophysiological biomarkers. Specifically, we will attempt to induce the functional modification of the cortical components by exploiting the features of the mirror neuron system, demonstrating relevant clinical, kinematic, and neurophysiological changes after AOT. With our study, we also want to provide, for the first time in Italy, the AOT home-based program while assessing its feasibility and impact.

**Trial Registration:**

ClinicalTrials.gov NCT04047134; https://clinicaltrials.gov/ct2/show/NCT04047134

**International Registered Report Identifier (IRRID):**

DERR1-10.2196/42094

## Introduction

### Background

Stroke is a leading cause of death and one of the most common causes of long-term disability that interferes with good quality of life [1]. Currently, one of the major components of rehabilitation interventions is focused on disability reduction to achieve the functional motor goal [2]. In the last few years, to improve activities of daily living (ADL), new noninvasive strategies that have emerged as rehabilitative treatments have been added to conventional motor rehabilitation programs [3,4].

One such noninvasive strategy is the action observation treatment (AOT), supported by results collected through randomized controlled trials [5-10]. This new rehabilitation approach is based on the network properties of the mirror neuron system (MNS) [11-13]. AOT involves the dynamic process of observing purposeful actions with the intention of imitating and then practicing those actions.

Extensive research over the last 20 years on human MNS [14-16] showed its importance not only in action recognition [17] but also in understanding action intentions and other important social cognitive aspects [14,16]. Moreover, higher activation of temporoparietal networks was found when a goal-directed action was observed [18]. Enabling the patients to acquire new skills and potentially relearn movement patterns, AOT is involved in the motor learning process and could be a helpful adjunctive treatment to conventional therapy in upper limb motor rehabilitation. Indeed, AOT seems to be a useful rehabilitation strategy in addition to physical therapy for improving upper limb functions, especially in daily activities after stroke [19,20], alone or in association with motor imagery [21]. Finally, because it is also recruited in the damaged brain [22,23], the MNS is demonstrated to provide remarkable rehabilitative outcomes [7,24-27].

On the basis of the MNS properties able to activate the sensory-motor system through the observation of actions as well as the execution of the same actions, the AOT is proving to be an innovative rehabilitative approach for recovery of patients with stroke in chronic stage with upper limb impairment; several findings are present in scientific literature [5,23,27-31].

On the basis of these results, several clinical studies have been published in recent years, suggesting the effectiveness of AOT in improving motor recovery and autonomy in ADL [19,20,24,32]. However, a deeper knowledge of the behavior of the sensorimotor cortex during AOT seems to be essential. Recent studies have investigated cortical responses during the visualization of standardized actions by using 4 biomarkers primarily: related desynchronization and synchronization of alpha and beta rhythms (event-related desynchronization [ERD] and event-related synchronization [ERS]), beta brain coherence, and mu rhythm suppression [33-36].

In our previous work, we administered AOT treatment to a group of patients with stroke and observed the predictive value of ERD of electroencephalography (EEG) alpha rhythm (ie, 8-13 Hz) during rehabilitation tasks and at the end of the treatment, as a sign of neural plasticity. In addition, increased functional dexterity was found in patients with stroke in the subacute stage that had AOT compared with their peers who underwent a control treatment [8].

Recently, our research group conducted a pilot study to assess which type of ADL visual stimulus was the most effective in inducing motor excitability during action observation. EEG signals were recorded in 20 patients with stroke in chronic stage during the observation of task-oriented upper limb tasks (including self-care and feeding actions) and nonfinalized actions. The comparison of EEG rhythms between the 2 groups of motor actions (ie, task-oriented and non–task-oriented) revealed that an increased suppression of mu and beta bands occurred during the observation of the former category [37].

The procedures described in this protocol, including recruitment, assessments, and visual stimuli for motor action, have been tested in a single-group uncontrolled feasibility study. Preliminary analysis from a qualitative observation of a few collected data by using the proposed protocol showed desynchronization effects of all cerebral rhythms (especially of the beta rhythm) after the AOT treatment (ie, pretest vs posttest assessment). In addition, a few patients had alpha and beta rhythm desynchronization compared with a group of healthy participants.

The encouraging results obtained from clinical outcomes were confirmed by studies that included instrumental assessments, among others: AOT has an additional positive impact on modifying cortical activation patterns [9,26,38] and in improving upper limb kinematics [39].

Furthermore, in Italy, to the best of our knowledge, no evidence has been found related to the effectiveness of home-based AOT programs compared with the same AOT performed in the hospital.

According to the inspiring results reported in the literature, AOT rehabilitative treatment exemplifies a translational step from neuroscience to clinical rehabilitation applications.

### Aims

The project aims to achieve the following objectives: (1) induce functional modification of the cortical components underpinning the action organization, exploiting the peculiar features of the MNS and demonstrating the relevant clinical, kinematic, and neurophysiological changes after AOT—in particular, the analysis of the AOT effectiveness will also consider the predictive value of neurophysiological biomarkers; and (2) provide the home-based AOT program, to the best of our knowledge, for the first time in Italy, thereby rendering evidence-based considerations regarding the feasibility and impact of our home-based AOT program for future systematic application of the approach.

## Methods

### Overview

A randomized, parallel-arm, controlled, outcome measurer–blinded, and multicenter clinical trial conducted in 2 neurorehabilitation centers in Italy aims to investigate the effectiveness of AOT in patients with chronic stroke (ie, >6 months from the first event) using quantitative EEG biomarkers, confirming the translational power of a tailored treatment.

A total of 60 individuals who have been enrolled will be randomized to the experimental group in hospital (EG@Hosp), experimental group at home (EG@Home), or control group (CG) in a 1:1:1 ratio.

The EG@Hosp will observe and execute ADL that are task-oriented actions (AOT protocol); the EG@Home will observe and perform the same ADL at home (AOT protocol at home); and the CG will not observe any motor task videos (but will watch video only on landscapes and historical issues), and after verbal instructions, they will perform the same actions observed by the other 2 groups.

After completing the patient screening, randomization will be performed before the first treatment session (T0). At baseline (T0), midintervention (after the 7th session; T1), and after the intervention (15th session or 5th week; T2), outcomes (ie, clinical scales, EEG and electromyography [EMG] signals, and kinematic parameters) will be assessed to evaluate impairment and functional abilities.

For each group, the total duration of the intervention will be 5 weeks (3 times a week) for a total of 15 sessions. After the end of the intervention, a 2-month follow-up assessment period (T3) will be scheduled to assess the maintenance and long-term effects.

The primary outcome assessed at T2 will be assessed by the Fugl-Meyer Assessment-Upper Extremity (FMA-UE) scores.

The trials will be performed at 2 neurorehabilitation centers in Italy: IRCCS San Raffaele Roma Hospital in Rome and at Casa di Cura del Policlinico Hospital in Milan.

All the data will be recorded in electronic case report forms through the REDCap (Research Electronic Data Capture; Vanderbilt University) system, accessed on the web via the internet for data collection and management.

**Figure 1:**
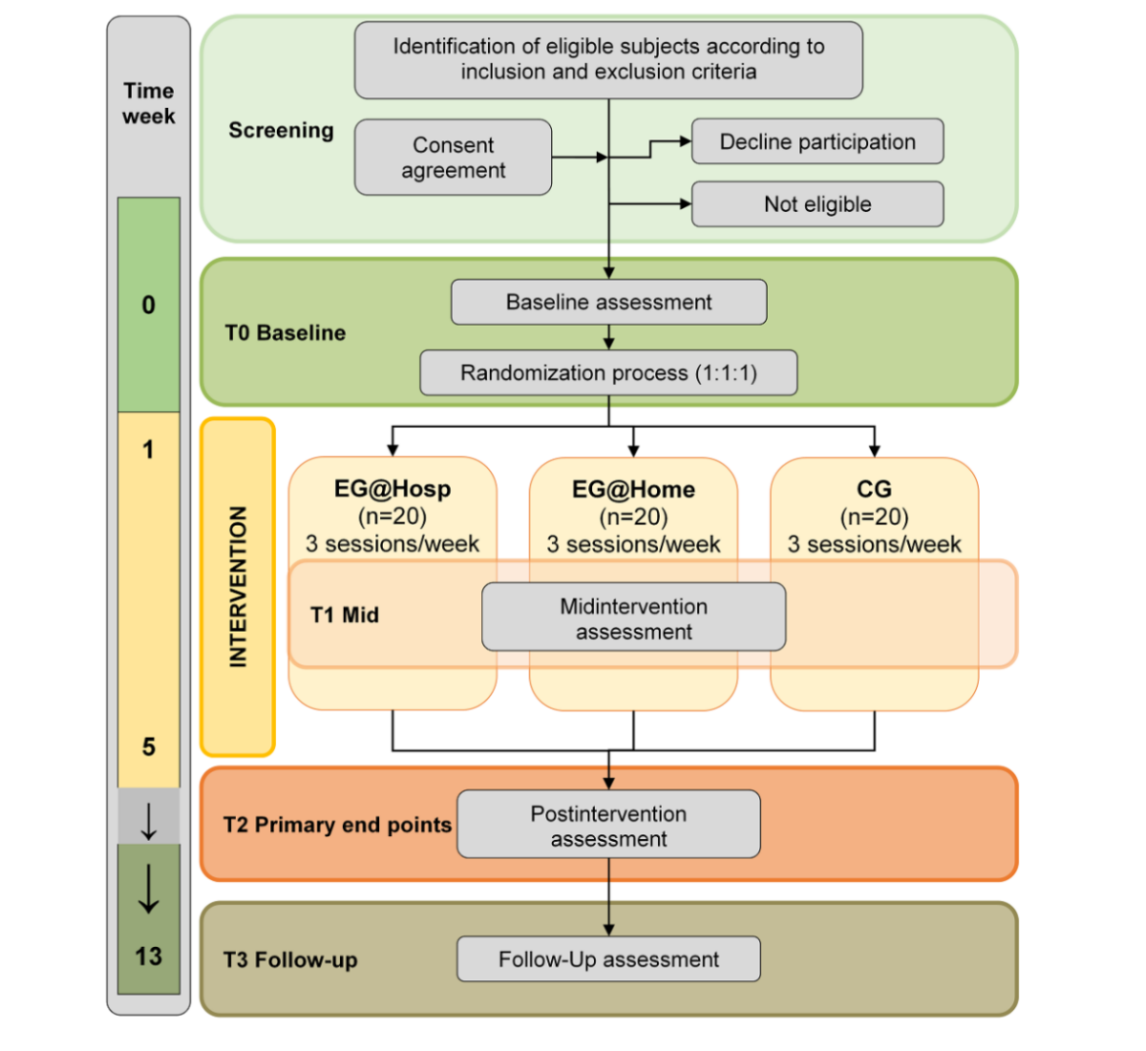
Flowchart of the proposed randomized controlled trial according to the Consolidated Standards of Reporting Trials. CG: control group; EG@Home: experimental group at home; EG@Hosp: experimental group in hospital.

A specific route diagram is presented in Figure 1. The protocol for this study was developed in accordance with the Standard Protocol Items: Interventional Trial Recommendations guidelines (SPIRIT) [40].

### Study Population, Setting, and Recruitment

This study will be conducted in patients with chronic stroke. Between January 2022 and October 2022, a total of 60 outpatients or inpatients were recruited from the 2 centers mentioned earlier. The intervention will be provided in 2 different settings: rehabilitation centers (EG@Hosp and CG) and the patient’s home (EG@Home).

Patients will be included after signing a written informed consent form, provided the inclusion and exclusion criteria are met.

**Figure 2:**
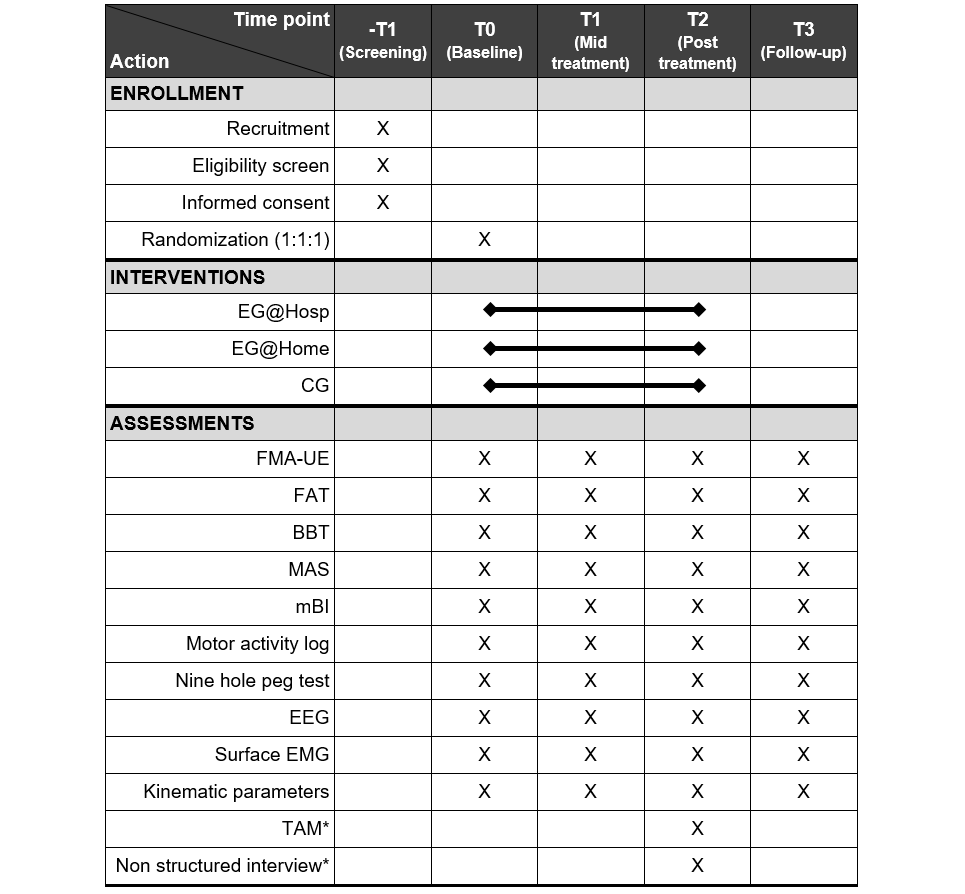
Standard Protocol Items: Interventional Trial Recommendations (SPIRIT) diagram describing schedule of enrollment, interventions, and assessments. *Only for experimental group at home intervention group. BBT: Box and Block Test; CG: control group; EEG: electroencephalography; EG@Home: experimental group at home; EG@Hosp: experimental group in hospital; EMG: electromyography; FAT: Frenchay arm test; FMA-UE: Fugl-Meyer Assessment-Upper Extremity; MAS: Modified Ashworth Scale; mBI: Modified Barthel Index; TAM: Technology Acceptance Model.

The schedule of enrollment, interventions, and assessments are presented in Figure 2.

### Selection Criteria

The inclusion and exclusion criteria of participants is shown in Textbox 1.

Patients with intellectual disability or other serious pathological conditions (eg, aphasia and hemianopsia) that affect their ability to watch or understand the proposed video were excluded.

**Participant inclusion and exclusion criteria.**

**Inclusion criteria**

- First ever unilateral ischemic stroke in the chronic stage (ie, the time elapsed from the event>6 months) provoked a clinically evident upper limb or hand deficit that affects the daily activities
- Diagnosis verified by brain imaging (magnetic resonance imaging)
- Aged 18-85 years
- Cognitive function sufficient to understand the experimental instructions (Mini-Mental State Examination Score of≥24)
- Chedoke-McMaster Stroke Assessment Scale—arm-hand section score of>1

**Exclusion criteria**

- Bilateral impairment
- Severe sensory deficits in the paretic upper limb
- Cognitive impairment or behavioral dysfunction
- Any other neurological or orthopedic deficit that affects arm function
- Any severe cardiovascular disease that can preclude intervention
- Any other current severe medical problems
- Refusal or inability to provide informed consent

### Randomization

On the enrollment day, by using the electronic data capture system software (ie, REDCap) and the method of central stratified regional group randomization, the enrolled patients will be randomly allotted to one of the 3 groups (ie, EG@Hosp, EG@Home, and CG).

### Blinding

Participants and intervention delivery facilitators cannot be blinded to group allocation. Evaluators conducting the clinical, neurophysiological (EEG and EMG), and physical (kinematics) assessments will be blinded to group allocation.

### Interventions

#### Overview

For all groups of participants (ie, EG@Hosp, EG@Home, and CG), the intervention will include 15 sessions spanning 5 weeks (3 sessions per week) in addition to the conventional motor rehabilitation program expected for each patient’s condition. Motor rehabilitation will be based on the relevant ADL that include at least one of the following: feeding, self-care, or external actions on the affected side. A total of 5 different videos (with the same motor task) will be presented in each session, beginning with the easiest action and ending with the most complex action. Each session will last approximately 15 minutes and will be repeated twice a day, at least 60 minutes apart.

#### AOT Protocol for EG@Hosp

Participants will be asked to carefully observe the videos showing different daily actions. Each motor act will be presented for 2 minutes. At the end of each motor act presentation, participants will execute the observed motor sequence for 1 minute (without any verbal instructions) with the affected upper limb.

Participants enrolled in EG@Hosp will perform rehabilitation treatment in the hospitals’ premises.

The video will be played on a 15-inch monitor at a distance from the participant such that there will be sufficient space to perform the required movements. During the repetition of the motor action, a black screen will appear on the monitor.

#### AOT Protocol for EG@Home

After appropriate training of patients and caregivers, the use of tablets will allow home-based treatment. In particular, a tablet will be enabled with a web-based program that will be used to train the patients (and receive feedback on their progress).

Participants enrolled in EG@Home will be asked to carefully observe the videos showing different daily actions on the tablet. The web-based program will present each motor act for 2 minutes. At the end of each motor act presentation, the web-based program will ask the participants to execute the observed motor sequence for 1 minute (by audio or video message) with the affected upper limb.

Participants enrolled in EG@Home will perform rehabilitation treatment at their homes.

A 7-inch tablet with Android software 11.0 will be used for the experimentation. Before handing the tablet to the patient, the researcher will schedule the AOT program via the web, defining the days when the patients should perform the treatment independently at home. Patients can check the days on which treatment will be performed by consulting the web-based application, specifically the section where the scheduling of sessions is provided, on the tablet. The research teams will be remotely monitoring the progress of the EG@Home sessions using a dedicated web platform.

The web-based application was developed by a specialist following the researchers’ specification.

#### CG Protocol

The participants will be asked to observe video clips with no content on human motor activities. Videos will concern scientific, geographical, and historical issues.

For EG@Hosp and EG@Home, 5 different video clips (each 2 minute long) will be presented during each session. At the end of each video, and after the therapist’s verbal instructions, participants will execute the motor task actions for 1 minute with the affected upper limb.

In this way, both the groups and controls will undergo the same amount of motor practice and receive the same amount of visual stimulation, with the only difference being the visual stimulus content.

Participants enrolled in the CG will perform rehabilitation treatment in the hospitals’ premises.

The video will be played on a 15-inch monitor at a distance from the participant such that there will be sufficient space to perform the required movements. During the repetition of the motor action, a black screen will appear on the monitor.

### Assessment: Instrumental Setup

The assessment setup and protocol will be the same as those used in similar studies [33,41] for the evaluation of impaired upper extremity functionality. The participant will sit in front of a target panel (eg, a 24-inch touch screen monitor) to perform the pointing task evaluation test.

The panel will have 9 targets; 8 of these, arranged in a circle, will be positioned 20 cm from its center, that is, the ninth target. The center of the target panel will be aligned with the shoulder acromion of the impaired upper limb. The distance between the participant and the center of the panel will be set according to each participant’s arm length measured with the fist closed. During the pointing task trials, the participants will be instructed to reach, at their self-selected speed, one of the 9 targets on the board.

All the participants enrolled in this study will perform assessment tasks in the hospitals’ premises.

### Assessment: Primary and Secondary End Points

#### Overview

A thorough assessment of the clinical improvement in the functionality of the affected upper extremity will be performed using the FMA-UE (primary outcome), which will be combined with other clinical scales (secondary clinical outcomes).

In addition, the secondary instrumental outcomes (ie, EEG, EMG, and kinematics) will be included to verify neural plasticity and motor recovery using neurophysiological and kinematic parameters.

In addition, only for home-based treatment (EG@Home) group, the usability of the platform and the participants’ satisfaction with the services will be evaluated using a specific questionnaire (Technology Acceptance Model [TAM]) and a nonstructured interview.

The variables related to the secondary instrumental outcomes (ie, EEG, EMG, and kinematics) will be evaluated during the standardized pointing task described earlier.

All the data will be analyzed using MATLAB software (MathWorks Inc). Specifically, the EEG recordings will be preprocessed offline using the MATLAB’s toolbox called *EEGLAB* for artifact removal.

#### The Functionality of the Affected Upper Extremity

To perform a thorough assessment, different motor components of the affected upper extremity will be evaluated using the FMA-UE combined with 6 other scales.

1. The FMA-UE [34,35] (primary outcome) is a stroke-specific, performance-based impairment index. It is designed to assess motor function, balance, sensation, and joint function in patients with poststroke hemiplegia.
2. The Frenchay Arm Test [36] is a measure of upper extremity proximal motor control and dexterity during ADL performance in patients with impairments resulting from neurological conditions. The Frenchay arm test is an upper extremity–specific measure of activity limitation.
3. The Box and Block Test [42] assesses the unilateral gross dexterity of the hand. It requires participants to move as many blocks as possible from one side to the other in 1 minute.
4. The Modified Ashworth Scale [43] measures resistance during passive soft tissue stretching and is used as a simple measure of spasticity of all movements of the different joints of the upper extremity.
5. The Modified Barthel Index [44] is an ordinal scale used to measure ADL performance. Each performance item is rated on this scale, with a given number of points assigned to each level or ranking. It uses 10 variables to describe ADL and mobility.
6. The Motor Activity Log [45] assesses the quantity (*amount* subscale) and quality (*how well* subscale) of the use of the upper limb during ADL performance.
7. The Nine Hole Peg Test [46] measures hand dexterity. This requires participants to repeatedly place and then remove 9 pegs into 9 holes, one at a time, as quickly as possible.

#### Neurophysiological Biomarkers

The following biomarkers will be used in the study:

1. The EEG signal will be recorded (0.01-100 Hz band pass; sampling frequency: 1024 Hz) using 64 to 128 electrodes (high-density EEG, system 10-10 increased) during the pointing task (described earlier). Alpha (8-13 Hz), beta (14-30 Hz), and mu (8-13 Hz) bandwidths will be registered. EEG biomarkers will be ERD and ERS of alpha and beta rhythms, beta brain coherence, and mu rhythm suppression [47-50].
2. Surface EMG signals from the activation of 2 pairs of agonist and antagonist muscles from 4 upper arm and shoulder muscles (brachial biceps, brachial triceps, anterior deltoid, and posterior deltoid) will be recorded. Dual Ag-AgCl snap electrodes with an interelectrode spacing of 2 cm will be used during the acquisitions. A standard procedure, in accordance with Surface ElectroMyoGraphy for the Noninvasive Assessment of Muscles recommendations [51], will be used for skin preparation and electrode placement. The reference electrode will be placed over the electrically neutral lateral wrist epicondyle. The outcome of this analysis will be the cocontraction index calculated during the initial portion (epoch) of the single pointing movement (forward and return movement will be analyzed separately), which is identified as the acceleration phase of the hand kinematics.

#### Kinematics

Kinematic parameters of the pointing motor task will be acquired at 100 Hz by using a 7-camera motion capture system (Vicon, Oxford Metrics Ltd). Spherical reflective markers for motion tracking will be placed on specific body landmarks. In addition, an inertial measurement unit will be placed over the metacarpophalangeal joint of the middle finger.

The data acquired during the assessment trial with respect to the hand trajectory (ie, metacarpophalangeal joint of the middle finger) will be evaluated using the following metrics, related to the entire turn [52]:

1. Number of peaks of the speed profile: If a point-to-point reaching movement has a low number of peaks, it means that few acceleration and deceleration periods are present [53].
2. The smoothness described by Teulings’s index is the rate of change of the acceleration in a movement [54]; a lower value of Teulings’s index indicates a smoother movement.
3. Movement accuracy will be evaluated using the normalized path length parameter, as described by Colombo et al [55]; essentially, it is the line obtained by normalizing the effective length path with the ideal path; when this parameter approximates 1, the movement accuracy is very high.
4. The absolute hand path error, as computed by Franklin et al [56], is the area between the actual movement path and the straight line; this is considered an index of learning, and a reduction of this metric indicates a better adaptation to the required task.

#### Usability of the Platform (Only for Home-Based Treatment)

The feasibility of the home-based treatment and the usability of the platform, as well as the participants’ satisfaction with the services, will be assessed by patients at the end of the intervention (T2) with the following scales:

1. TAM [57] includes 3 criteria (perceived ease of use, perceived usefulness, and behavioral intention to use). A Likert scale has been developed in which the user will rate from 1 to 7 whether they agree with a series of statements.
2. Nonstructured interviews (adapted from the TAM) to investigate patients’ feelings.

### Sample Size

As the changes in the effects of upper limb AOT in patients with stroke on EEG biomarkers have not been previously investigated, we have estimated the sample size based on previously published studies [58,59], comparing AOT and conventional therapy. We estimated the sample size required to detect differences in the effects of “group”×“time” interactions on primary clinical outcome (FMA-UE).

For this outcome, an effect size of 0.495 [60,61] is expected based on previous studies. Given the expected effect size, a total sample size of 54 will be required for repeated ANOVA with a power of 0.8 and a 2-sided type-I error of 0.05 (number of groups=3 [ie, EG@Hosp, EG@Home, and CG]; number of measurements=3 [ie, T1, T2, and T3]). Therefore, when considering a dropout rate of 10%, we plan to recruit 20 participants for each group (a total of 60 participants) for this study.

### Statistical Analysis

#### Baseline Comparability Analysis

The baseline characteristics of the participants will be summarized and compared. These include the descriptions of demographic data, symptoms, and general conditions. The 2-tailed *t* test or nonparametric statistical method will be used for quantitative data. Categorical data will be analyzed using chi-square or Fisher exact tests and the Wilcoxon test.

#### Analysis of the Effectiveness

Analysis of the primary outcome (ie, functionality of the affected upper extremity) will be performed using the per-protocol principle. Treatment effects will be compared using a 2-way repeated ANOVA for clinical measurements, considering “time” (3 levels: T0, T1, and T2) as a within-subject factor and “group” (3 levels: EG@Hosp, EG@Home, and CG) as a between-subject factor.

In addition, the percentage of participants who passed the minimal clinically important differences of the FMA-UE will be compared using chi-square tests between the 3 groups.

The mean power across the assessment “time” (ie, T0 and T2) related to the action observation condition will be implemented in a mixed-design ANOVA to evaluate significant changes. A separate ANOVA will be performed to assess power changes in the stimuli selection data, using “treatment” (ie, EG@Hosp vs EG@Home vs CG) as the between-subjects factor and action as the within-subjects factor. To assess the predictive value of biomarkers for AOT effectiveness, an analysis of covariance (ANCOVA) will be performed considering the clinical data as the dependent variable, the “group” as a fixed factor, and the biomarkers as covariates.

Subsequently, to evaluate kinematic parameters recovery, a mixed design ANOVA will be implemented with “time” and “treatment” as within- and between-subjects factors, respectively.

The level of significance will be set at *P*<.05. For post hoc comparisons, the level of significance will be set at *P*<.017 after Bonferroni adjustment (.05/n; the number of comparisons, n=3) for the comparison of interaction effects. Cohen *d* will be calculated to determine the effect size of the change in scores for the behavioral motor outcomes between the groups. Immediate training effects (data from T0-T2) and the durability of training effects (data from T2-T3) will be separately investigated using mixed effect models.

Clinical-neurophysiological correlations will also be analyzed: the correlation between the ERD and factors such as the clinical scale score will be studied. To evaluate the differences in trends related to the clinical evolution of the patients, we will define a new variable to group the participants according to the presence or absence of relevant clinical improvements. The relevant clinical improvement will be defined by a clinical specialist for this purpose.

### Data Collection and Management

The study data will be collected and managed using REDCap electronic data capture tools hosted at the IRCCS San Raffaele Roma Hospital and Casa di Cura del Policlinico Hospital [62,63]. REDCap is a secure web-based software platform designed to support data capture for research studies, providing (1) an intuitive interface for validated data capture, (2) audit trails for tracking data manipulation and export procedures, (3) automated export procedures for seamless data downloads to common statistical packages, and (4) procedures for data integration and interoperability with external sources.

### Ethics Approval

The ethics committees of both involved centers (ie, IRCCS San Raffaele Roma Hospital and Casa di Cura del Policlinico Hospital) reviewed and approved the study protocol (Ethics Committee IRCCS San Raffaele Roma: protocol code RP05/2018; Ethics Committee Milano Area 2: protocol 705_2018bis).

The institutional review boards of both centers approved the study and will receive study reports at the middle and end of the study and monitor the study implementation and data collection.

Any deviations from the protocol will be promptly notified to the ethics committees and will be applied only after approval.

This study will be conducted in accordance with the principles of the Declaration of Helsinki. Written informed consent forms will be obtained from each participant before the start of the study. Only those participants who provided informed written consent before starting will be included. During the study, written data will be stored in a closed cabinet; all data, after anonymization, will be entered into a REDCap electronic data capture repository. The input data will be double-checked by another research assistant. Personal data will be discarded after 5 years.

The results of this study will be disseminated through peer-reviewed journals and national and international academic conferences only by the professionals directly involved in the clinical trial.

### Patient Withdrawal Criteria

Patients will be informed of the possibility of interrupting the study at any time they deem appropriate. Any interruption will be fully documented in the case report form by the investigators. The investigators shall make all reasonable efforts to maintain the patients who express a willingness to interrupt the participation in the study protocol.

Patients may prematurely discontinue the study if any of the following conditions occur:

1. Request from the patient, even if not motivated
2. Withdrawal of consent by the patient to participate in the study
3. Modification of the psychophysical state of the patient, significantly altering at least one of the variables considered in the inclusion or exclusion criteria
4. The occurrence of serious adverse reactions, which in the opinion of the investigator, make the continuation of the study impossible
5. Absence from >3 consecutive experimental session
6. Inability of the patient to continue the study for organizational and personal reasons

## Results

Participants will be identified from hospital records or among those who show interest in participating by seeing the initiative through the communication channels of the hospitals, posters, and networks; all of them will be contacted by phone. After obtaining permission, the physicians will visit and explain the study. If they agree, the research assistants will manage their recruitment. This will be performed every week to identify participants.

The results of the evaluations will be released to the participants upon request. The results of this study will be presented at national and international patient organizations and to the general public.

The study protocol is part of a project (project code GR-2016–02361678) approved and funded by the Italian Ministry of Health.

## Discussion

### Rationale

The rationale of this study protocol is based on the AOT in patients with chronic stroke for upper limb rehabilitation. AOT relies on the MNS network to boost motor functions in patients affected by motor impairments, engaging the brain networks active during action execution. The imitation of observed gestures may increase the reorganization of the primary motor cortex, contributing to the formation of motor memories of the observed action, thus improving the physiological processes underlying motor learning [28,30].

It is well known that the MNS plays an essential role in considering the actions of others and in our ability to learn by imitation [64]. Furthermore, the activation of motor areas by AOT seems to be reinforced by the subsequent active execution of observed actions [9]. Indeed, as a motor representation technique, AOT has become an emerging motor learning training strategy to improve motor recovery in different healthy and pathological populations. Moreover, AOT preactivates specific areas of the brain, reinforcing intact cortical networks and facilitating the activation of the damaged ones [65]. Recently, a systematic review on this topic highlighted how AOT is an effective method for improving upper limb motor functions after stroke and that task-based AOT (based on goal-oriented activities, eg, a reaching task) might be superior to movement-based AOT (eg, a pure movement of elbow extension without any functional goals), as mirror neurons are more receptive to object-related actions [24]. The authors concluded that the optimal dosage, substantiality of effects, and underlying neural mechanisms of AOT in improving upper limb motor functions in patients with stroke should be considered in further investigations [24]. According to previous findings, we believe that increasing evidence supporting the effectiveness of observation-based inventions to assist traditional therapeutic practice in the rehabilitation of motor disorders [66] must be explored.

On the basis of these findings, the proposed study protocol aims to identify the effects of AOT and motor rehabilitative treatment on the clinical and functional status of patients with stroke through an advanced multidomain assessment and to infer the effects on brain plasticity.

This study will be a starting point for the evaluation of the effectiveness of the 2 AOT scenarios proposed (ie, AOT in the hospital and AOT at home) in patients with chronic stroke (ie, the time elapsed from the event is >6 months), as well as for the assessment of the predictive value of neurophysiological biomarkers.

Specifically, we will attempt to induce functional modification of the cortical components by exploiting the features of the MNS, demonstrating relevant clinical, kinematic, and neurophysiological changes after AOT.

The added value of a multidomain assessment that includes test and clinical scales and kinematic and neurophysiological (ie, EEG and EMG) outcomes aims to identify neurophysiological biomarkers capable of evaluating the effectiveness of AOT and confirming the translational power of a tailored rehabilitation treatment for chronic stroke.

Moreover, as AOT is involved in the motor learning process and could be a useful addition to conventional therapy, an innovative home-based AOT program will be implemented in the framework of this protocol study. Indeed, the continuity of care and personalized rehabilitation for people who are affected by chronic diseases is often interrupted after transitioning from the hospital to the home environment [67].

### Future Implications

As a result of the COVID-19 pandemic, telerehabilitation and home care are growing rapidly, offering opportunities for integrating digital health interventions into community-based aging services. The COVID-19 pandemic has accelerated the adoption of technology and virtual care in patients’ homes, and this trend likely to stay after the pandemic. Although notable age differences in technology use remain, the adoption of key technologies by older adults is the new challenge. With this purpose, this study protocol will permit the investigation of whether a home-based program could be effective, especially in terms of exercise capacity and perceived ease of use, offering comparable benefits to hospital-based programs.

The translational research results will ensure advances in the optimization and personalization of the rehabilitative process, both in hospitals and at home, thus improving upper limb motor functions and the quality of life of patients with chronic stroke.

## Abbreviations

ADL: activities of daily living
ANCOVA: analysis of covariance
AOT: action observation treatment
CG: control group
EEG: electroencephalography
EG@Home: experimental group at home
EG@Hosp: experimental group in hospital
EMG: electromyography
ERD: event-related desynchronization
ERS: event-related synchronization
FMA-UE: Fugl-Meyer Assessment-Upper Extremity
MNS: mirror neuron system
REDCap: Research Electronic Data Capture
SPIRIT: Standard Protocol Items: Interventional Trial Recommendations guidelines
TAM: Technology Acceptance Model

## Data Availability

The data sets generated and analyzed during this study will be available from the corresponding author upon reasonable request.
